# Nontraumatic Acute Elevation of Pancreatic Enzymes following Percutaneous Nephrolithotomy: A Rare Complication

**DOI:** 10.1155/2017/7430328

**Published:** 2017-11-15

**Authors:** Nikolaos Ferakis, Antonios Katsimantas, Georgios Zervopoulos, Vasileios Klapsis, Spyridon Paparidis, Filippos Venetsanos, Konstantinos Bouropoulos

**Affiliations:** Department of Urology, Korgialenio-Benakio Hellenic Red Cross Hospital, Athanasaki 1, 115 26 Athens, Greece

## Abstract

Herein, we report the case of a 48-year-old female who developed nontraumatic acute pancreatitis following left supracostal Percutaneous Nephrolithotomy. Three hours postoperatively, the patient developed fever with signs and symptoms consistent with hydrothorax, which was confirmed radiologically and was managed conservatively. The following days, the patient developed manifestations of Systemic Inflammatory Response Syndrome with epigastric pain, nausea, and vomiting. Blood, urine, and sputum cultures were negative. Serum amylase and lipase levels were elevated 3 and 13 times above the normal level, respectively. Imaging studies revealed no pathologic findings from pancreas. These findings were consistent with the diagnosis of acute pancreatitis.

## Introduction

The most common postoperative complications associated with Percutaneous Nephrolithotomy (PCNL) are fever/sepsis, bleeding, urinoma, perinephric hematoma, urinary leakage, and injury to adjacent viscera [1–4]. Acute pancreatitis (AP) is a common clinical entity, but its development following PCNL is extremely uncommon.

## Case Report

**Figure 1:**
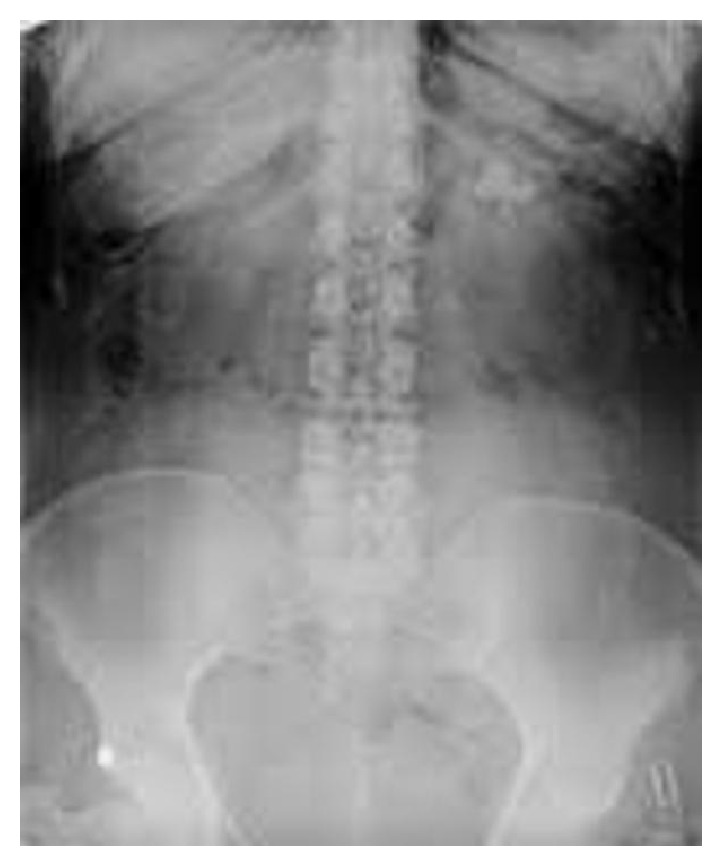
Preoperative plain X-ray of the kidney, ureter, and bladder region showing a partial staghorn left renal stone.

A 48-year-old female was admitted to our department because of a partial staghorn renal stone at the upper pole of the left kidney (Figure 1). Her medical history included hypertension, obesity, and uterine cancer hysterectomy one year ago. Preoperative urine culture was negative.

The patient underwent PCNL in prone position under general anesthesia. Following placement of a 6 Fr ureteral catheter in lithotomy position and injection of contrast agent, a single tract at the upper pole calyx was created above the 12th rib under fluoroscopy. After tract dilation with a balloon dilator, a 30 Fr Amplatz sheath was positioned inside the calyx of puncture. Following stone fragmentation with a pneumatic lithotripter, the stones were removed using grasping forceps and an 18 Fr nephrostomy tube was inserted for postoperative drainage. The patient was stone-free and there were no intraoperative complications. Stone analysis revealed struvite stone.

**Figure 2:**
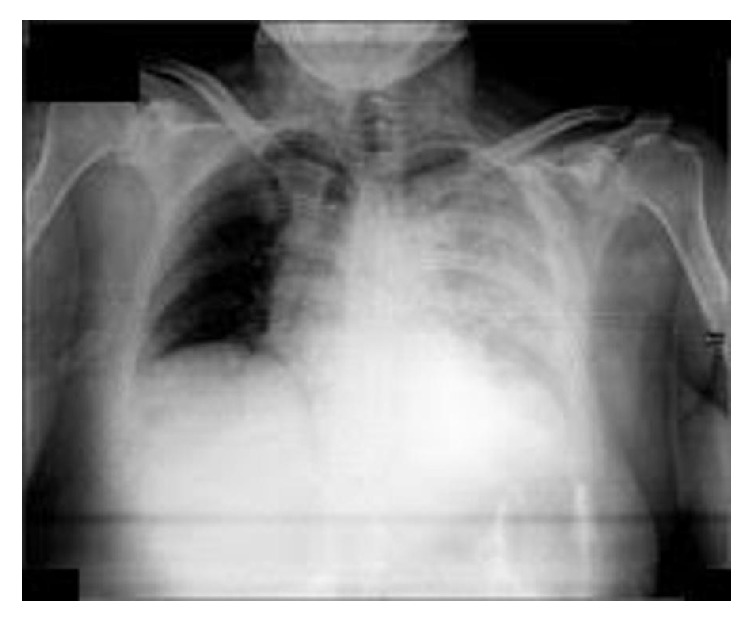
Chest X-ray (three hours postoperatively) demonstrating left pleural effusion.

Three hours postoperatively, the patient complained of pain at the left hemithorax and developed respiratory distress. Her vital signs included blood pressure of 80/40 mmHg, heart rate of 97 beats/min, respiratory rate of 25 breaths/min, temperature of 38°C, and oxygen saturation of 88% on room air. Hemoglobin value was 12.4 g/dl. Chest X-ray showed left pleural effusion (Figure 2). Computed tomography (CT) scan demonstrated left hydrothorax with contrast material, small left pneumothorax, and postoperative alterations at the area of the left kidney. These manifestations were attributed to the infection stone and pleural injury. For the pleural effusion, the patient was managed conservatively, according to the thoracic surgeon's recommendation, and demonstrated progressive improvement clinically and radiologically.

**Figure 3:**
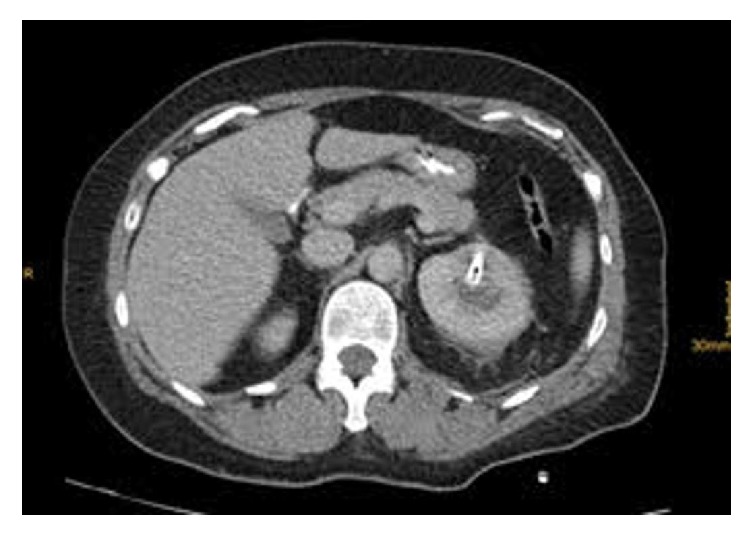
Abdominal CT scan (fourth postoperative day) demonstrating no pathologic findings from pancreas and nephrostomy tube in the left renal pelvis.

The following days, the patient developed manifestations of Systemic Inflammatory Response Syndrome (SIRS). Temperature was greater than 38°C and heart rate was permanently greater than 90 beats/min. White Blood Cell (WBC) count was 12.6 × 10^3^/*μ*L and 3.3 × 10^3^/*μ*L at the first and fourth postoperative days, respectively. Blood pressure, respiratory rate, and oxygen saturation were in the normal range. Antibiotic treatment with piperacillin-tazobactam was administered empirically. C-reactive protein value was 196 mg/L (normal < 3 mg/L). Blood, urine, and sputum cultures were negative. On the fourth postoperative day, the patient developed epigastric pain radiating to the back, nausea, and vomiting. Serum amylase value was 313 U/l (normal: 28–100 U/l) and serum lipase value was 798 U/l (normal: 10–60 U/l). Serum levels of calcium, triglyceride, and liver transaminases were in the normal range. Serum bilirubin value was 1.73 mg/dl (normal: 0.2–1.2 mg/dl) and serum alkaline phosphatase value was 247 U/l (normal: 32–104 U/l). Abdominal ultrasonography and magnetic resonance (MR) cholangiopancreatography were normal. Chest and abdominal CT scan demonstrated minor left hydrothorax and postoperative alterations at the area of the left kidney. There were no pathologic findings from the abdominal viscera (Figure 3). These findings were consistent with the diagnosis of AP. According to the internal medicine consultation, the patient was treated with fluid resuscitation, pain control, complete bowel rest, and replacement of piperacillin-tazobactam by meropenem trihydrate and vancomycin hydrochloride. The patient demonstrated complete progressive recovery and was discharged on the fourteenth postoperative day.

## Discussion

PCNL is a well-established treatment option for patients with large, multiple, or inferior calyx renal stones [1–4]. Supracostal puncture is a safe and effective choice in a selected group of patients [3]. Parameters that influence the complication rate are the surgeon's experience, the operative time, the stone's size and opacity, the number of punctures/tracts, and the presence of bacteria within the stone [1, 3, 4].

AP following PCNL is extremely rare. Chitale et al. presented a case of AP following right PCNL, where they noted mild increase of serum amylase levels, with pathologic findings on CT scan [2]. AP was considered to be reactionary or sympathetic [2]. Osman et al. reported their experience with 315 PCNL treatments, where one patient developed AP postoperatively [4]. AP was not attributed to the procedure and the authors pointed out that there were no injuries in the surrounding organs [4].

The diagnosis of acute pancreatitis demands the presence of two of the following criteria: (a) abdominal pain consistent with the disease, (b) serum amylase and/or lipase greater than 3 times of the upper limit of normal, and/or (c) characteristic findings on CT or MR imaging [5]. Our patient met two criteria out of them.

The diagnosis of SIRS demands the presence of two of the following criteria: (a) heart rate > 90 beats/min, (b) respiratory rate > 20 breaths/min, (c) temperature > 38.3 or <36°C, and/or (d) WBC count > 12 × 10^3^/*μ*L or <4 × 10^3^/*μ*L [6]. We observed that our patient met SIRS criteria for several days postoperatively.

The most common causes of AP are gallstones and alcohol abuse [2, 5, 7]. Other causes include medication, infectious agents, hypercalcemia, hyperparathyroidism, hypertriglyceridemia, abdominal trauma, endoscopic retrograde cholangiopancreatography, and benign or malignant masses that obstruct the pancreatic ducts [2, 5, 7]. There are cases of AP in which an etiology cannot be established [5]. These cases are characterized as idiopathic [5].

We need to mention that increased serum levels of pancreatic enzymes are common in critically ill patients with multiorgan dysfunction because of ischemia, inordinate inflammatory response, oxidative stress, cellular apoptosis, and/or metabolic derangement [8]. This increase is not accompanied by clinical and imaging features of pancreatitis in many cases [8]. In our case, we cannot preclude that the elevation of pancreatic enzymes was reactionary because of SIRS, as CT scan did not demonstrate pathologic findings from pancreas and SIRS preceded the pancreatitis appearance. However, the elevation of serum amylase and lipase 3 and 13 times above the normal range, respectively, was accompanied by symptoms of AP and we did not observe clinical or serological signs of multiorgan dysfunction. Moreover, the appearance of pancreas on CT scan may be normal in mild AP, as in our case, and the instructions for the treatment of AP, according to the internal medicine consultation, led, as a result, to the complete progressive patient's recovery [7]. We believe that our patient developed nontraumatic, idiopathic, mild AP, which complicated the postoperative course.

AP may mimic other clinical entities and must be managed immediately and aggressively because of its possible complications. In conclusion, when a patient develops signs and symptoms of AP following PCNL, the urologist should exclude this clinical entity.

## References

[1] Seitz C., Desai M., Häcker A. (2012). Incidence, prevention, and management of complications following percutaneous nephrolitholapaxy. *European Urology*.

[2] Chitale S., Mbakada R., Burgess N. (2005). Pancreatitis following percutaneous nephrolithotomy. *Scandinavian Journal of Urology*.

[3] Sekar H., Krishnamoorthy S., Kumaresan N., Ramanan V. (2016). Supracostal punctures for PCNL: Factors that predict safety, success and stone free rate in stag horn and non-stag horn stones: A single centre experience and review of literature. *Journal of Clinical and Diagnostic Research*.

[4] Osman M., Wendt-Nordahl G., Heger K., Michel M. S., Alken P., Knoll T. (2005). Percutaneous nephrolithotomy with ultrasonography-guided renal access: experience from over 300 cases. *BJU International*.

[5] Tenner S., Baillie J., Dewitt J., Vege S. S. (2013). American college of gastroenterology guideline: management of acute pancreatitis. *The American Journal of Gastroenterology*.

[6] Thomas B. S., Jafarzadeh S. R., Warren D. K., McCormick S., Fraser V. J., Marschall J. (2015). Temporal trends in the systemic inflammatory response syndrome, sepsis, and medical coding of sepsis. *BMC Anesthesiology*.

[7] Koo B. C., Chinogureyi A., Shaw A. (2010). Imaging acute pancreatitis. *British Journal of Radiology*.

[8] Chaari A., Hakim K. A., Bousselmi K. (2016). Pancreatic injury in patients with septic shock: A literature review. *World Journal of Gastrointestinal Oncology*.

